# Cross-Cultural Adaptation and Psychometric Evaluation of the Arabic Clinical Reasoning Scale Among Nursing Students

**DOI:** 10.3390/nursrep16070214

**Published:** 2026-06-25

**Authors:** Minimole Kalarickal Kunjan, Avudaippan Seethalakshmi, Zechariah Jebakumar Arulanantham, Sethuraman Nagalakshmi

**Affiliations:** 1 Department of Nursing Foundation, Sri Ramachandra Institute of Higher Education and Research, Porur, Chennai 600116, India; 2 Nursing Department, Prince Sultan Military College of Health Sciences, Dhahran 31932, Saudi Arabia; 3 Department of Nursing Foundation, Faculty of Nursing, Sri Ramachandra Institute of Higher Education and Research, Porur, Chennai 600116, India; seethalakshmi.a@sriramachandra.edu.in; 4 Scientific Research and Publication Department, Vice Deanship of Postgraduate Studies and Scientific Research, Prince Sultan Military College of Health Sciences, Dhahran 31932, Saudi Arabia; zechariah@psmchs.edu.sa; 5 Department of Pharmaceutics, Sri Ramachandra Faculty of Pharmacy, Sri Ramachandra Institute for Higher Education and Research (DU), Porur, Chennai 600116, India; nagalakshmi.s@sriramachandra.edu.in

**Keywords:** clinical reasoning, nursing students, cross-cultural adaptation, psychometric validation, Arabic version, clinical reasoning scale

## Abstract

**Background:** Clinical reasoning is a vital competency for safe nursing practice, yet no validated Arabic instrument exists to assess this skill among nursing students in Saudi Arabia. **Aim:** This study aimed to translate, culturally adapt, and psychometrically evaluate the Arabic version of the Clinical Reasoning Scale (CRS) and to investigate clinical reasoning among Saudi nursing students. **Methods:** This methodological instrument validation study with a cross-sectional survey component was conducted in Saudi Arabia between January 2024 and May 2025 among nursing students. The Arabic Clinical Reasoning Scale (CRS-A) was translated and culturally adapted in accordance with the WHOQOL Group guidelines for instrument translation. Content validity was assessed by 10 experts, and construct validity was evaluated using exploratory factor analysis (*n* = 365). The response rate was 98.65%. Internal consistency was evaluated using Cronbach’s alpha (*n* = 365), and test–retest reliability (*n* = 30) was measured with the Intraclass Correlation Coefficient (ICC) over a two-week period. Descriptive statistics, one-way analysis of variance (ANOVA), and independent sample *t*-tests were also performed. **Results:** The tool’s content validity (S-CVI = 0.98) was confirmed by a panel of experts. The CRS-A demonstrated excellent temporal stability (ICC = 0.95, *p* < 0.001) and internal consistency (Cronbach’s α = 0.935). The exploratory factor analysis showed that the 16 items’ factor loadings ranged from 0.542 to 0.807, and three factors accounted for 64.33% of the total variance. Students self-reported agreement with clinical reasoning abilities (mean scores: 3.81–4.18). No significant differences in clinical reasoning were found by age (*p* = 0.102) or gender (*p* = 0.226), but significant differences were found by Cumulative Grade Point Average (CGPA) (*p* < 0.001). **Conclusions:** The Arabic Clinical Reasoning Scale demonstrated preliminary psychometric performance for measuring clinical reasoning among Arabic-speaking student nurses. It provides educators with a valuable tool for identifying learning needs and evaluating educational interventions.

## 1. Introduction

Decision-making skills are crucial in nursing management as they involve assessing information, problem-solving, and critical reasoning. Clinical Reasoning (CR) is an essential nursing competency that enables nurses to collect patient information, interpret clinical cues, identify patient problems, make informed decisions, and evaluate patient outcomes. Effective clinical reasoning plays a major role in healthcare safety outcomes, effective patient care, and evidence-driven nursing practice [1]. In nursing education, the development of clinical reasoning skills is considered a critical outcome, as nursing students are expected to apply theoretical knowledge and clinical judgment in increasingly complex healthcare settings [2]. By contrast, poor clinical decision-making accounts for more than half of clinical errors, underscoring a critical relationship between nurses’ cognitive competence and patient well-being [3]. Furthermore, reputable professional organizations, including the National Council of State Boards of Nursing [4] and the American Association of Colleges of Nursing (AACN) [5], emphasize CR as a necessary and crucial nursing skill. In complex healthcare situations, which are frequently marked by rapidly changing patient needs, technological advancements, and an abundance of healthcare information, the significance of clinical reasoning among nurses is further heightened [6,7,8].

More than 10,000 Saudi nurses remain diploma-prepared, necessitating accelerated pathways to baccalaureate completion through bridging programs [9]. Moreover, in recent years, the Kingdom of Saudi Arabia (KSA) has undergone transformative developments, specifically in nursing education and healthcare delivery, driven by its national Vision 2030 healthcare transformation agenda [10]. An enhancement of the clinical competencies of the nursing workforce is expected as a result of a shift in professional standards. Considering this, studies conducted in KSA have stressed the importance of assessing students’ CR abilities to identify learning needs, which will guide the selection of effective teaching strategies to improve clinical competence and patient care outcomes [9,11,12,13].

The findings of Al-Thubaity [12] indicated a statistically significant difference in perceptions of clinical decision-making between undergraduate and bridging program participants, with those in bridging programs demonstrating superior decision-making skills. The aforementioned findings raised important questions about the adequacy of clinical reasoning development within standard baccalaureate curricula. Dickison et al. [4] also suggested that over half of newly qualified Saudi nurses working in pediatric settings could not correctly identify all clinical scenarios presented. In this regard, Huang et al. [14] found that 23% of newly graduated nurses are unable to identify changes in patient condition, and 54% cannot effectively manage patient problems. As per Bae et al. [1], effective clinical reasoning skills are associated with high-quality nursing care, patient well-being, and overall positive patient outcomes. The importance of clinical reasoning among nurses is especially heightened in complex healthcare environments, often characterized by rapidly changing patient needs, technological innovations, and an explosion of healthcare information [6]. Considering this, the present study aimed to translate, culturally adapt, and psychometrically evaluate the Arabic version of the Clinical Reasoning Scale (CRS) and to investigate clinical reasoning among Saudi nursing students.

Recent nursing literature continues to show that clinical reasoning, clinical decision-making, and clinical competency are related yet distinct concepts. Clinical reasoning is the cognitive process of gathering, interpreting, and integrating patient data to understand a situation [15]. Clinical decision-making is the selection of the most appropriate action based on clinical reasoning, professional knowledge, and patient needs [16]. Clinical competency is more comprehensive and refers to the nurse’s overall ability to apply knowledge, skills, attitudes, judgment, and professional behaviors to provide safe and effective care [17]. Thus, clinical reasoning is the cognitive basis for clinical decision-making, and both clinical reasoning and decision-making are key to developing and demonstrating overall clinical competency in nursing practice.

Clinical reasoning is a complex cognitive process that uses formal and informal thinking strategies to gather and analyze patient data, evaluate their significance, and inform the weighing of alternative actions and clinical suggestions. It encompasses metacognitive awareness and professional competence, highlighting its multidimensional nature [8]. Jessee [18] suggested that clinical reasoning requires integrating theoretical knowledge with experiential learning, which, in turn, facilitates the development of nursing education and practice. According to Guandalini et al. [19], to meet both professional and ethical requirements, nurses must have clinical reasoning skills before entering practice settings. The capacity for sound clinical reasoning is widely recognized as the cornerstone of safe and effective nursing practice [20].

Clinical reasoning and clinical judgment have been conceptualized and assessed using a variety of models, each with its own limitations. Benner’s novice-to-expert model describes the developmental progression of clinical competence from novice to expert but does not provide a structural framework for teaching clinical reasoning [21]. For instance, the work of Tanner in 2006 [22] conceptualizes reasoning across four phases, namely, noticing, interpreting, responding, and reflecting, thereby forming the basis of instruments such as the Lasater Clinical Judgment Rubric. In light of this, Levett-Jones et al. [23] developed a clinical reasoning cycle emphasizing the iterative process of cue collection, information processing, problem identification, and outcome evaluation, encompassed by the five rights of clinical reasoning. The present study was guided by Huang et al.’s framework of clinical reasoning competencies for nursing students, which conceptualizes clinical reasoning as a dynamic and continuous process that evolves in response to changes in the clinical context. The framework comprises four interrelated domains: awareness of clinical cues, confirmation of clinical problems, determination and implementation of actions, and evaluation and reflection. Together, these domains represent essential competencies for effective clinical reasoning and provide a theoretical foundation for assessing nursing students’ clinical reasoning abilities [14,24].

Various instruments have been developed to measure clinical reasoning and clinical judgment in nursing students. One of the most common instruments used to measure nursing students’ clinical judgment is the Lasater Clinical Judgment Rubric (LCJR) developed by Lasater [25] based on Tanner’s model [22]. This tool has 11 behavioral dimensions distributed across four domains: noticing, interpreting, responding, and reflecting. Each dimension is rated on a four-level developmental scale ranging from beginning to exemplary. The use of the construct, however, is based on faculty observation and requires rater training to attain inter-rater reliability [26]. Other methods, such as the Script Concordance Tests and Key Feature Questions, are frequently tailored to particular educational settings and subject areas [27,28]. Therefore, their use in evaluating global clinical reasoning processes in nursing students may be limited.

Among the instruments available, the CRS developed by Huang et al. [24] was specifically designed to assess clinical reasoning competence among nursing students. The scale was developed based on the framework of competencies in clinical reasoning for nursing students developed by Huang et al. in 2018 [14] and validated in a sample of 1504 nursing students enrolled in three nursing education programs in Taiwan. The final instrument consists of four domains: awareness of clinical cues, confirmation of clinical problems, determination and implementation of actions, and evaluation and reflection. An important requirement identified in nursing education is the creation of an instrument specific to the nursing discipline and applicable in different nursing education settings [29]. Given the absence of a validated Arabic version, cross-cultural adaptation and psychometric evaluation of the CRS were warranted. Compared with other tools, CRS is concise, easy to administer, demonstrates satisfactory content validity and construct validity (as assessed by confirmatory factor analysis), and shows internal consistency reliability, and is used in the current study [24].

The recent cross-cultural validations also showed similar psychometric performance. Bayzat and Dinc [30] translated the clinical reasoning evaluation simulation tool into Turkish and found that it had a CVI of 0.91 and a Cronbach’s alpha of 0.88, with acceptable fit indices (GFI = 0.911 and RMSEA = 0.042). The Persian Clinical Reasoning Competency Scale was validated by Bae et al. (2023) with 1100 Iranian nurses, yielding an overall alpha of 0.89 and factor loadings ranging from 0.62 to 0.88 [1]. Findings from these recent cross-cultural validations, with acceptable psychometric performance, verified the feasibility of cross-cultural adaptation and underscored the importance of rigorous validation procedures tailored to linguistic and cultural settings.

Despite the growing importance of clinical reasoning assessment, the utility of CRS within the Arabic-speaking cohort remains constrained by the absence of a culturally adapted, psychometrically validated Arabic version [31], underscoring the need for rigorous validation procedures tailored to linguistic and cultural settings. To address this gap, the objectives of the current investigation were as follows: (1) translating and cross-culturally adapting the Clinical Reasoning Scale (CRS) developed by Huang et al. [24] into Arabic; (2) evaluating the initial psychometric properties of the instrument such as the content validity, temporal stability, internal consistency, and construct validity; and (3) assessing clinical reasoning levels among nursing students using the adapted scale. Having a culturally adapted Arabic version of the CRS would provide nursing educators and researchers with a standardized tool to measure clinical reasoning among nursing students in Arabic-speaking educational settings.

## 2. Materials and Methods

### 2.1. The Research Design

A methodological instrument validation study with a cross-sectional survey component was used to translate, culturally adapt, and test the psychometric properties of the Arabic version of the Clinical Reasoning Scale (CRS-A) among nursing students.

### 2.2. Sample Size and Setting

#### Setting

This study was conducted in the nursing department at Prince Sultan Military College of Health Sciences (PSMCHS), Saudi Arabia. The nursing program was delivered on a semester basis. A consecutive sampling method was employed for a conveniently available population of nursing students at PSMCHS to recruit the required sample size who met the inclusion criteria and agreed to participate in the study. To achieve the required sample size, students from three consecutive semesters were invited to participate in the psychometric evaluation of the CRS-A. Recruitment proceeded until the required sample size was reached. Requirements for inclusion were as follows: (1) baccalaureate nursing students, enrolled in medical and surgical nursing clinical courses; (2) students in the second and third years of study; (3) students who were able to read and understand Arabic; and (4) students who voluntarily agreed to participate and provided informed consent. The exclusion criteria were baccalaureate nursing students not registered in the selected medical and surgical nursing clinical courses. This study was conducted between January 2024 and May 2025.

In the program’s curriculum, the medical nursing course is offered in the second year of study, and the surgical nursing course in the third year. The clinical courses in medical–surgical nursing were chosen specifically because the study was focused on assessing clinical reasoning ability in the nursing management of patients with medical and surgical disorders. These students had already completed basic nursing courses and were exposed to theoretical instruction, nursing skills laboratory training, simulation-based learning activities, and supervised clinical practice experiences through medical and surgical nursing courses. These learning experiences provided opportunities to develop and apply clinical reasoning skills in patient assessment, clinical decision-making, and nursing management, and therefore constituted an appropriate population for evaluating the CRS-A. First-year students were excluded because they had not yet taken the medical and surgical nursing courses required to develop the clinical reasoning skills related to patient management. Students in the fourth year were excluded because they were studying specialized advanced courses unrelated to the course of study.

The sample size for the exploratory factor analysis was determined according to established guidelines recommending a minimum of participants-to-item ratio of 10:1 [32]. Since the CRS-A has 16 items, a minimum sample size of 160 participants was needed with a ratio of 10:1 of participants to items. To ensure adequate power for the psychometric evaluation and to allow for potential non-response, 370 nursing students were recruited. A total of 5 participants did not complete the questionnaire, leaving a final sample of 365 for analysis, yielding a response rate of 98.65%. This study is reported according to the STROBE guidelines for cross-sectional studies, ensuring transparent and comprehensive reporting of the study methods and findings.

### 2.3. Clinical Reasoning Scale (CRS) Survey Tool

The current study adapted the Clinical Reasoning Scale (CRS) developed by Huang et al. in 2023 [24]. The scale was developed in Taiwan to assess CR competence among nursing students across different nursing education programs, addressing the lack of a nursing-specific instrument for this purpose. The instrument was based on the competencies framework for CR for nursing students by Huang et al. and was the first in Chinese. Content Validity (CV) was established through a two-round Delphi study with experts in nursing education, and construct validity was established through Confirmatory Factor Analysis (CFA). Data for the validation study were collected from 1504 final-semester nursing students in 10 universities in Taiwan. The final CRS comprises 16 items across four categories: awareness of clinical cues (4 items), confirmation of clinical problems (4 items), determination and implementation of actions (4 items), and evaluation and reflection (4 items). Each item is rated on a 5-point Likert scale, where 1 represents “strongly disagree,” and 5 represents “strongly agree.” The scale exhibited strong psychometric properties, with content validity indices ranging from 0.85 to 1.00, Cronbach’s alpha ranging from 0.78 to 0.89 across subscales, and construct validity with model fit indices as follows: GFI = 0.97; AGFI = 0.95; RMSA = 0.049. The final model with four factors explained 49.03% of the variance in CR competence. With its theoretical grounding and robust psychometric performance, CRS is an optimal tool for assessing clinical reasoning among nursing students [24].

In the first section of the tool, participants completed the demographic data comprising age, gender, and Cumulative Grade Point Average (CGPA). For analytical purposes, CGPA was categorized in line with the institutional grading system into excellent (4.75–5), very good (3.75–4.74), good (2.75–3.74), and satisfactory (2–2.74).

### 2.4. Translation and Cultural Adaptation

Although English is the medium of instruction in the nursing program, Arabic is the native language of all students. Therefore, the CRS was translated, cross-culturally adapted into Arabic, and psychometrically evaluated to improve linguistic appropriateness, enhance item comprehension, and facilitate accurate measurement of clinical reasoning within the context of Saudi nursing education. The original authors granted permission to use and translate the CRS. The translation and cultural adaptation of the CRS were performed following the guidelines of The WHOQOL Group [33] for the translation of instruments. The process involved forward translation of the original English version into Arabic, independently performed by two bilingual translators from a qualified translation company (C.R.2051253177) who were proficient in both Arabic and English. One of the translators had a background in nursing and was familiar with the concepts being measured, while the other was an expert in language translation. An expert panel reviewed the translated version to assess semantic and cultural equivalence. After deliberation and agreement among the study team, the Arabic version of the Clinical Reasoning Scale is confirmed. After that, the backward translation was carried out by two independent bilingual translators who were blinded to the original version of the instrument. This process ensured that the translation was linguistically accurate and conceptually equivalent to the original. An expert panel of nursing educators and bilingual specialists compared the backward translations with the original CRS to identify discrepancies. Before the CRS-A was finalized for psychometric evaluation, a pilot study of the pre-final Arabic version was conducted with 30 nursing students to assess its clarity, readability, cultural appropriateness, and comprehensibility. The same participants completed the instrument again after 2 weeks to assess test–retest reliability. These steps were taken to attain conceptual, semantic, and cultural equivalence between the English and Arabic versions of the instrument.

### 2.5. Psychometric Evaluation of the CRS-A

The CRS-A’s preliminary psychometric qualities were examined after translation, with a focus on the Content Validity (CV), construct validity, internal consistency reliability, and test–retest reliability. This evaluation was guided by the COSMIN (Consensus-based Standards for the selection of Health Measurement Instruments) recommendations [34].

The content validity of the CRS-A was evaluated by a panel of 10 experts in nursing academia and professional practice. Professionals rated the items for relevance on a 4-point scale between “not relevant” (1) and “highly relevant” (4). Prior to calculating the Content Validity Index (CVI), expert ratings were dichotomized: ratings of 3 or 4 were considered relevant and coded as 1, and ratings of 1 or 2 were considered not relevant and coded as 0. The I-CVI was calculated as the proportion of experts who rated an item as relevant [35]. The tools’ Item-wise Content Validity Index (I-CVI) > 0.78, Scale-wise Content Validity Index by the average method (S-CVI/Ave) > 0.90, and Scale-wise Content Validity Index by the universal agreement method (S-CVI/UA) > 0.80 were acceptable [36].

Exploratory factor analysis was performed by using the principal component method and Varimax rotation. The minimum acceptable value for a factor loading was 0.3. Sampling adequacy was assessed using the Kaiser–Meyer–Olkin (KMO) test, with values greater than 0.5 considered acceptable. Barlett’s sphericity test was also used to test the appropriateness of the model. The factor number was determined by the scree plot and eigenvalues greater than 1 [37].

Thirty second-year baccalaureate nursing students at a nursing college in the Eastern Province of KSA were selected via a purposive sampling technique, and the participants completed the CRS-A with a two-week interval under standardized conditions. This interval was selected based on recommendations for health measurement instruments, as it is long enough to minimize recall bias but short enough to preclude meaningful changes in the underlying construct being measured and thus offers an appropriate assessment of temporal stability [38]. A questionnaire was distributed by hand to the recruited participants. The study’s purpose and confidentiality, as well as the voluntary nature of participation, were all conveyed to students, and informed consent was obtained prior to data collection. Test–retest stability was assessed using the Intraclass Correlation Coefficient (ICC); ICCs ≥ 0.75 were considered acceptable [39].

Internal consistency was assessed using data from 365 nursing students who completed the CRS-A. The internal consistency of the overall scale and its four subscales was assessed using Cronbach’s alpha, with values ≥ 0.70 considered acceptable [40]. 

The English version of the Clinical Reasoning Scale (Supplementary File S1) and the Arabic version (Supplementary File S2) are available as Supplementary Materials.

### 2.6. Clinical Reasoning Assessment

To evaluate nursing students’ clinical reasoning skills, the tool was administered to 365 baccalaureate nursing students who were about to complete their medical and surgical nursing clinical courses. Students used the tool to assess their clinical reasoning skills in managing patients with various medical and surgical conditions. After explaining the purposes, procedures, and participants’ rights, the tools were distributed. The students took 15 min to complete the questionnaire. Participants provided informed consent prior to participating in the study. All participants were promised confidentiality, anonymity, and voluntary participation. Students were assured that their responses would not affect their academic performance. The collected information was processed and securely stored on a password-protected computer. Only the research team had access to the data.

### 2.7. Data Analysis Technique

To determine the CV of the CRS-A, the item-wise CVI and scale-wise CVI were calculated via the averaging method and the universal agreement method. To verify construct validity, an Exploratory Factor Analysis (EFA) was performed using principal component analysis and Varimax rotation in SPSS 31.0. The Kaiser–Meyer–Olkin (KMO) and Bartlett’s sphericity tests were performed, and eigenvalues, explanatory power, and loading values were calculated. Test–retest stability was assessed using the Intraclass Correlation Coefficient (ICC), and the internal consistency of the overall scale and its four subscales was assessed using Cronbach’s alpha. Descriptive statistics, including frequencies, percentages, means, and standard deviations, were used to analyze CRS-A and demographic data. Independent *t*-tests were used to compare CRS scores by gender, and a one-way ANOVA was used to examine associations between CRS scores and age groups and academic performance, as measured by cumulative grade point average (CGPA). A 95% confidence interval was used to assess statistical significance; *p* < 0.05 was the cutoff. The IBM SPSS, version 31.0 (IBM Corp., Armonk, NY, USA) was used to conduct the analysis.

### 2.8. Ethical Considerations

Permission to use the tool was obtained from the original author via email. The Institutional Review Board (IRB) at the institution where the study was conducted granted ethical approval (IRB-2024-NUR-006). All participants provided informed consent and participated voluntarily. Participant confidentiality and anonymity were preserved throughout the study, and the study complied with the Declaration of Helsinki’s ethical guidelines.

## 3. Results

### 3.1. Pilot Study Findings

In the pilot study with a sample size of 30, the participants reported that the items were clear, easily understood, and culturally relevant. The translated CRS-A did not pose any interpretive or completion difficulties, and no modifications were made to the translation. Preliminary reliability testing suggested excellent internal consistency for the entire scale (Cronbach’s α = 0.97).

### 3.2. Background Variables

A total of 365 nursing students participated in the study. Most participants were in the 18–23 years’ age group (49.0%), followed by 24–29 years (26.3%), 30–35 years (17.0%), and 36+ years (7.7%). The sample consisted of 81% female participants and 19% male participants. Participants were asked to report their cumulative grade point average (CGPA). According to the institutional grading system, the CGPA is graded as excellent (4.75–5), very good (3.75–4.74), good (2.75–3.74), and satisfactory (2–2.74). The majority of students reported that their CGPA falls in the very good range (60%), followed by good (26.6%), excellent (11.5%), and satisfactory (1.9%).

### 3.3. Content Validity

With the S-CVI/Ave being 0.98, the S-CVI/UA being 0.85, and the I-CVI being from 0.9 to 1.0, as shown in Table 1, the CRS-A met the acceptability criteria. Items 4 and 8 had I-CVI scores of 0.90, and all other items had I-CVI scores of 1.00. One expert gave a lower rating, suggesting replacing one Arabic term in each item with more appropriate language to improve linguistic clarity and cultural relevance. We included these suggestions in the translated version.

### 3.4. Construct Validity

To explore the underlying factor structure of the 16-item CRS-A, we conducted an exploratory factor analysis (EFA) using principal axis factoring with Varimax rotation. The correlation matrix showed significant positive correlations among all items (*p* < 0.001; range = 0.257 to 0.968). The determinant of the correlation matrix was 1.164 × 10^−5^, indicating no severe multicollinearity [41].

**Figure 1:**
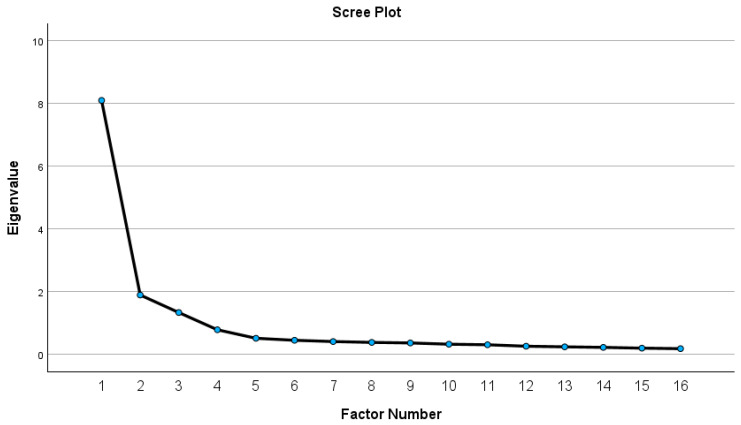
Scree plot for factor extraction.

The Kaiser–Meyer–Olkin (KMO) measure of sampling adequacy was 0.925, indicating excellent sampling adequacy. Bartlett’s test of sphericity was statistically significant (χ^2^ = 4065.39, *p* < 0.001), indicating that the data were suitable for factor analysis. In addition, the anti-image correlation matrix revealed that the Measures of Sampling Adequacy (MSA) values ranged from 0.895 to 0.950, suggesting that all items were appropriate for inclusion in the analysis [42]. Using the Kaiser criterion (eigenvalues > 1), three factors with eigenvalues of 1.0 or greater were extracted. The three factors accounted for 70.84% of the total variance, as indicated by the first eigenvalues. The three-factor solution accounted for 64.33% of the total variance after extraction. The three factors explained 23.16%, 20.75%, and 20.42% of the variance after Varimax rotation, respectively (Table 2). Inspection of the scree plot (Figure 1) indicated a clear break after the third factor, supporting retention of a three-factor structure [43].

The communalities after extraction ranged from 0.551 to 0.748, indicating that the extracted factors explained 55.1% to 74.8% of the variance of individual items. The communalities exceeded the suggested minimum of 0.40, justifying the retention of all items [43]. Finally, the Varimax technique was used to determine which items loaded onto which factors, resulting in 16 items loading onto three factors (Table 3).

The rotated factor matrix showed factor loadings from 0.542 to 0.807 for the items. Factor 1 included eight items (items 5–12); factor 2, four items (items 13–16); and factor 3, four items (items 1–4). The rotation converged in eight iterations and yielded a stable, interpretable factor structure. The results support the construct validity of the CRS-A and its multidimensional structure among nursing students. The naming of the EFA-identified factors reflected each factor’s content. The first factor was named “confirmation of clinical problems, determination and implementation of action plans.” The second factor was labeled “evaluation and reflection,” and the third factor was labeled “awareness of clinical cues.” There were some cross-loadings among a few items across two factors, especially items 5, 6, 11, and 12. However, all items had primary loadings > 0.50 and were retained in the factor solution.

### 3.5. Test–Retest Reliability (ICC)

The ICC, based on a two-way mixed-effects model with absolute agreement, was used to determine the stability of the CRS-A over a two-week period; the single-measures ICC was 0.95 (95% CI: 0.896–0.975; *p* < 0.001), indicating excellent reliability for single administrations of the CRS-A. The average ICC was 0.97 (95% CI: 0.945 to 0.987; *p* < 0.001), indicating excellent test–retest reliability for the average of repeated measurements. The higher average measures ICC reflects greater stability and precision achieved by averaging scores across repeated administrations (Table 4).

### 3.6. Internal Consistency

Cronbach’s alpha was used to evaluate the CRS-A’s internal consistency. With a sample size of 365, the entire scale showed good internal consistency (Cronbach’s α = 0.935). The Cronbach’s alpha coefficients for the awareness of clinical cues, confirmation of clinical problems, determination and implementation of action plans, and evaluation and reflection domains in the CRS-A were 0.883, 0.886, 0.886, and 0.896, respectively, indicating good internal consistency across all domains of the instrument. Reliability coefficients for all domains were higher than the recommended cut-off of 0.70 [40], indicating high interrelatedness of items within each domain and that the domains reliably measured the intended construct. These findings provide strong support for the reliability of the Arabic version of the CRS.

Responses to the 16 items in the CRS-A are recorded on a 5-point Likert scale, ranging from “Strongly Disagree” (1) to “Strongly Agree” (5). The Arabic version of the instrument includes the same items and structure as the original English version, which ensures consistency in the measurement of CR competency across languages.

### 3.7. Descriptive Statistics of the Arabic Clinical Reasoning Scale Items

Table 5 presents the averages for all 16 items, ranging from 3.81 to 4.18 on a 5-point Likert scale, with 4 representing agreement and 5 representing strong agreement. These values indicate that nursing students self-rate their clinical reasoning as in agreement with all competencies.

Table 6 illustrates the domain-wise scores of the CRS-A. The highest mean scores (M = 4.15) were observed in the evaluation and reflection category, and the students agreed most strongly that they can reflect on the steps involved in solving problems (M = 4.18) and re-evaluate unresolved patient problems (M = 4.18). The lowest mean scores were observed in the confirmation of clinical problems area (M = 3.86), especially in the collection of data prior to confirming health problems (M = 3.81). Even though these scores do not leave the range of agreement, they indicate that the students find confirming clinical problems to be their least developed competency. Students’ scores in the awareness of clinical cues domain and the determination and implementation of action plans domain were 4.08 and 3.99, respectively.

### 3.8. Comparison of Clinical Reasoning Scores with Background Variables

#### 3.8.1. Comparison of Clinical Reasoning Scores by Gender

Male and female participants’ clinical reasoning scores did not differ significantly, as indicated by a *t*-test (t(363) = 1.21, *p* > 0.05). The mean score of clinical reasoning among male nursing students was M = 65.86 (SD = 10.11), while the mean score of female nursing students was M = 64.12 (SD = 7.80) (Table 7). These findings show that CR levels are similar in males and females.

#### 3.8.2. Comparison of Clinical Reasoning Scores by Age Group

ANOVA was used to compare the clinical reasoning scores of the four age groups, as shown in Table 8.

The highest mean CRS score was observed in the 18–23 age group (M = 65.26; SD = 8.28; *n* = 179), followed by the 30–35 group (M = 64.03; SD = 6.97; *n* = 62), the >35 group (M = 63.86; SD = 6.80; *n* = 28), and the 24–29 group (M = 62.76; SD = 8.47; *n* = 96). However, the ANOVA revealed no statistically significant difference in clinical reasoning scores across age groups (F(3, 361) = 2.08, *p* = 0.102). These results reveal that clinical reasoning levels among nursing students of different ages are similar.

#### 3.8.3. Comparison of Clinical Reasoning Scores by Academic Performance

The ANOVA was also used to compare clinical reasoning scores across four CGPA categories: Excellent, Very Good, Good, and Satisfactory. The comparison showed a statistically significant difference in CRS scores across CGPA categories (F(3, 361) = 6.19, *p* < 0.001). The overall mean CRS score for students in the Good CGPA category was the lowest (M = 61.33; SD = 8.33), whereas students in the Excellent category had the highest mean score (M = 65.55; SD = 7.37). Table 9 confirms a significant difference in CRS scores by academic performance, with students with higher CGPAs likely to report higher CR competency.

The CGPA category had a small effect on overall clinical reasoning scores (η^2^ = 0.049; 95% CI: 0.011–0.092), accounting for approximately 4.9% of the variance in CRS scores due to differences in academic performance. Turkey’s post hoc comparisons showed that the clinical reasoning scores of students with excellent and good CGPA were significantly different (MD = 4.21, *p* = 0.021). Similarly, CR scores of students with very good and good CGPA were significantly different (MD = 3.97, *p* < 0.001). No significant differences were found among the remaining CGPA categories.

## 4. Discussion

The current study translated, culturally adapted, and psychometrically tested the CRS-A among 365 nursing students in Saudi Arabia.

In the pilot study, alpha values greater than 0.95 may indicate potential item redundancy [44]. However, the EFA conducted with a sample size of 365 revealed a stable three-factor structure, suggesting that the items measured related but distinct dimensions of clinical reasoning. The increased alpha in this study may be attributable to the sample’s relative homogeneity, the instrument’s cultural and linguistic adaptation, and the high item correlations after translation, which may have improved item clarity and consistency. Therefore, the high reliability coefficient is likely to reflect the coherence rather than the duplication of item content. Moreover, the internal consistency was further evaluated during the psychometric evaluation.

The cultural adaptation and translation process produced a linguistically equivalent tool, and an acceptable CV was achieved through the review of 10 nursing experts. The analysis of the CV showed item-wise CVI values ranging from 0.9 to 1.0, and the S-CVI was 0.98 on average, with both considered substantial [45]. These results are similar to the original scale, as Huang et al. (2023) reported that the item-CVI of the CRS ranged from 0.85 to 1.0 and that the scale CVI was 0.98 [24].

The EFA results supported the measure’s suitability for assessing the clinical reasoning of Arabic nursing students. The EFA results reveal that the 16 items were adequately correlated for factor analysis, with a KMO test score of 0.925 and Bartlett’s test of sphericity (*p*-value < 0.001). The Varimax method yielded three factors, retaining 16 items. On examining each item, items 5, 6, 7, 8, 9, 10, 11, and 12 loaded onto factor 1; items 13, 14, 15, and 16 loaded onto factor 2; and finally, items 1, 2, 3, and 4 loaded onto factor 3. The moderate-to-strong correlations between the items and their factors provided evidence for the robustness of the three-factor method. Despite some cross-loadings, the principal loadings were much higher than the suggested level, indicating adequate factor discrimination. Results support the construct validity of the CRS-A and confirm its multidimensional structure among nursing students, and all items were retained. The factor names and their elements are presented in Table 10.

The first factor, “confirmation of clinical problems, with determination and implementation of action plans,” had the highest explanatory power, accounting for 50.63% of the total variance. The study emphasizes the significance of problem identification and clinical decision-making processes in the clinical reasoning of nursing students and shows that these competencies constitute a major dimension of the CRS-A construct. The second factor, “evaluation and reflection,” points to the need to evaluate patient outcomes and reflect on clinical decision-making as essential elements of clinical reasoning. The results indicate the importance of reflective practice for improving clinical judgment and decision-making among nursing students. The third aspect, “awareness of clinical cues,” highlights the ability to identify and understand important patient information. This component emphasizes the fundamental role of cue detection and information collection in the clinical reasoning process in facilitating timely, successful nursing interventions. In the current study, a three-factor structure accounted for 64.33% of the total variance, supporting the construct validity of the CRS-A. The results are generally consistent with those of Huang et al. (2023) [24], who found a multidimensional structure for both the original CRS and the final 16-item version, with four factors accounting for 49.03% of the variance.

Four factors were identified in the original study, and three in the present study. The difference between the present study and the original validation study may partly be explained by differences in participant characteristics. Validation was performed with students in the final semester of nursing education by Huang et al. (2023) [24], while validation was performed with second- and third-year students enrolled in medical and surgical nursing courses in this study. The psychometric properties may have been impacted by differences in educational level and clinical exposure, as clinical reasoning develops progressively with academic progression and clinical practice.

Test–retest reliability was also high, with an intraclass correlation coefficient of 0.95 (95% CI: 0.896–0.975, *p* < 0.001), indicating that the CRS-A is stable over time and can be used in research. With a Cronbach’s alpha of 0.935, the CRS-A demonstrated good internal consistency, suggesting that the items accurately assessed clinical reasoning in nursing students. The values exceed the minimum recommended threshold of 0.70 for acceptable reliability and indicate a high level of internal consistency among the scale items [40]. This value was higher than the original CRS developed by Huang et al. (α = 0.89) [24]. Similarly, the current study’s internal consistency value supports the Persian Clinical Reasoning Competency Scale validation, which also yielded high consistency among scale items with a total alpha of 0.89 [46]. The higher reliability coefficients observed in the current study may be due to the high inter-item correlations and consistent responses of Arabic-speaking nursing students.

Clinical reasoning is influenced by cultural, educational, and healthcare system contexts, so cross-cultural adaptation is necessary when an instrument developed in one setting is applied in another. Taiwan and Saudi Arabia differ in language, communication style, educational approach, and clinical training environment. Valid and reliable instruments adapted to the target culture and language are necessary for cross-cultural research and quality patient care. The original CRS, developed in Taiwan, includes four domains: awareness of clinical cues, confirmation of clinical problems, determination and implementation of actions, and evaluation and reflection. Huang et al. noted that the psychometric properties of the CRS might be influenced by the cultural background and suggested further validation studies in other cultural areas. During the adaptation process, the CRS items were checked for semantic, cultural, and conceptual equivalence. Minor linguistic changes were made to improve clarity and relevance for Arabic-speaking nursing students, while preserving the original meaning of the items. These adaptations were not only a literal translation of the language but also improved the cultural appropriateness and usefulness of the CRS-A. These procedures support the cross-cultural validity of the tool and address the original developer’s recommendation to evaluate the instrument in different cultural contexts. The developed instrument demonstrated strong psychometric properties and was found to be suitable for assessing the clinical reasoning of Arabic-speaking nursing students [24,47].

The descriptive results indicate that Saudi nursing students agreed with their clinical reasoning skills on all 16 items, with average values varying from 3.81 to 4.18 on a 5-point scale (4 = agree). The mean scores were highest in Domain 4 (evaluation and reflection in computing: M = 4.15), while Domain 1 (awareness of clinical cues: M = 4.08), Domain 3 (determination and implementation of actions: M = 3.99), and Domain 2 (confirmation of clinical problems: M = 3.86) had the lowest scores. This trend is quite similar to the original CRS validation research, which also found that validation of clinical problems was the least proficient among the three types of nursing programs in Taiwan [24]. This trend is maintained across various cultural and educational settings, suggesting that verifying clinical issues is a problematic area of clinical reasoning development in nursing education—likely a manifestation of the cognitive depth required to integrate a variety of clinical indicators and relate theoretical knowledge to practice [48].

The current study revealed no statistically significant difference in clinical reasoning scores between male (M = 65.86; SD = 10.11) and female (M = 64.12; SD = 7.80) nursing students (t(363) = 1.21, *p* > 0.05). This indicates that gender may not be a singular determinant in the cultivation of clinical reasoning in baccalaureate nursing students. These results align with the study by Kaur, G. [49], which found that socio-demographic factors, including gender, did not correlate significantly with clinical reasoning skills. These findings support the view that clinical reasoning may be influenced more by educational exposure, problem-solving skills, self-efficacy, and clinical practice experience than by gender.

The clinical reasoning scores did not differ significantly across age groups (F(3, 361) = 2.08, *p* = 0.102), indicating that clinical reasoning competency is similar among nursing students. The above conclusion indicates that clinical reasoning development is primarily a function of educational experiences rather than age, underscoring the importance of specific pedagogical interventions regardless of age. Conversely, significant differences were observed among the CGPA categories (F(3, 361) = 6.19, *p* < 0.001). The lowest mean CRS score was observed in the Good CGPA category (M = 61.34; SD = 8.33), and the higher academic performance categories were significantly higher. This aligns with the study by Xu and Wang [50], which found that scores in professional knowledge significantly affected clinical thinking ability among nursing undergraduates. The connection between academic performance and CR likely indicates the role of theoretical knowledge in clinical judgment. Students with excellent or very good CGPAs had significantly higher clinical reasoning scores than those with good CGPAs. However, the small effect size (η^2^ = 0.049) indicates that academic achievement, as measured by CGPA, has a small effect on clinical reasoning skills. Although academic achievement was associated with clinical reasoning ability, CGPA accounted for only a small portion of the variance in clinical reasoning. This finding suggests that while academic achievement may play a role in developing clinical reasoning, other factors, such as clinical exposure, critical thinking, learning experiences, and self-directed learning, may also contribute to its development.

The CRS-A validation offers nurse educators and researchers in Saudi Arabia and other Arab nations a valid and dependable tool in measuring clinical reasoning competency. The conclusion that confirmation of clinical problems is the weakest area indicates that nursing curricula should focus on systematic methods for training skills relevant to concept mapping, learning through simulation with structured debriefing, and reasoning through case analysis [47].

Several limitations of the current study should be noted.

First, the study was conducted with nursing students from a single educational environment in Saudi Arabia, which may limit the generalizability of the findings to Arab-speaking populations and healthcare education settings. Second, although the CVI, construct validity via EFA, internal consistency, and test–retest reliability provided preliminary evidence of the psychometric properties of CRS-A, Confirmatory Factor Analysis (CFA), testing of convergent and discriminant validity, assessment of criterion validity, and measurement invariance analysis were not performed. Third, social desirability bias may be introduced when subjective assessment measures are used, as students may exaggerate their clinical reasoning skills. Fourth, the cross-sectional design of the study limited the assessment of psychometric properties that require longitudinal follow-up, such as predictive validity, responsiveness to change, and longitudinal measurement variance. Finally, fourth-year nursing students were not included in the sample. As senior students typically have more extensive clinical exposure and advanced clinical reasoning skills, the findings may not be fully generalizable to all undergraduate nursing students.

Despite these limitations, CRS-A underwent rigorous translation and cultural adaptation procedures in accordance with accepted practices, including forward translation, expert panel review, back translation, a pilot study, and content validation by experts to ensure conceptual, cultural, and semantic equivalence. Second, the test–retest reliability test was conducted after 2 weeks, along with internal consistency measurement and exploratory factor analysis conducted on a sufficient sample size of 365, providing preliminary evidence of the psychometric quality of the Arabic Clinical Reasoning Scale. Third, this study is the first to test a comprehensive clinical reasoning tool among Arabic-speaking nursing students in Saudi Arabia. Although the CFA and measurement of invariance of the tool were not conducted in the current study, the results provide preliminary evidence of the CRS-A’s validity and reliability. This study provides a valuable contribution to nursing education research by offering a valid and reliable tool for measuring clinical reasoning competencies in Arabic-speaking baccalaureate nursing students, a population that has received limited attention in psychometric research.

Future studies should further evaluate the tool’s psychometric properties using CFA, convergent and discriminant validity, criterion validity, and measurement invariance analyses in larger, more diverse samples across different educational and healthcare settings to further confirm the cross-cultural validity and comparability of the CRS-A. To evaluate the instruments’ stability and predictive capacity across the various phases of nursing education, longitudinal studies are advised. Educational strategies, such as clinical reasoning training models, may also be integrated into nursing curricula to strengthen students’ clinical judgment, decision-making skills, and overall professional competence. Future studies should include students from multiple institutions and academic levels to increase the generalizability of the Arabic Clinical Reasoning Scale.

## 5. Conclusions

The Arabic version of the clinical reasoning scale demonstrated excellent test–retest reliability, acceptable content validity, good construct validity, and internal consistency. Thus, the instrument demonstrated preliminary psychometric properties and may be used to assess clinical reasoning among Arabic-speaking nursing students. Clinical reasoning levels among Saudi nursing students are moderately high, and confirmation of clinical problems is the weakest area. There were significant academic performance disparities in clinical reasoning scores but not in age and gender. The Arabic Clinical Reasoning Scale offers a key instrument for nurse educators and researchers in Saudi Arabia to assess educational interventions, identify learning needs, and facilitate the development of the nursing workforce in line with Saudi Vision 2030. However, further studies involving diverse samples and additional validation processes, including CFA and criterion validity testing, are recommended to further confirm its psychometric robustness.

## Figures and Tables

**Table 1:** Content Validity Index (CVI) of the Arabic version of the Clinical Reasoning Scale (CRS-A).

Items	Raters											Experts in Agreement	I-CVI	UA
	1	2	3	4	5	6	7	8	9	10				
Q1	1	1	1	1	1	1	1	1	1	1		10	1	1
Q2	1	1	1	1	1	1	1	1	1	1		10	1	1
Q3	1	1	1	1	1	1	1	1	1	1		10	1	1
Q4	1	1	1	1	1	1	0	1	1	1		9	0.9	0
Q5	1	1	1	1	1	1	1	1	1	1		10	1	1
Q6	1	1	1	1	1	1	1	1	1	1		10	1	1
Q7	1	1	1	1	1	1	1	1	1	1		10	1	1
Q8	1	1	1	1	1	1	0	1	1	1		9	0.9	0
Q9	1	1	1	1	1	1	1	1	1	1		10	1	1
Q10	1	1	1	1	1	1	1	1	1	1		10	1	1
Q11	1	1	1	1	1	1	1	1	1	1		10	1	1
Q12	1	1	1	1	1	1	1	1	1	1		10	1	1
Q13	1	1	1	1	1	1	1	1	1	1		10	1	1
Q14	1	1	1	1	1	1	1	1	1	1		10	1	1
Q15	1	1	1	1	1	1	1	1	1	1		10	1	1
Q16	1	1	1	1	1	1	1	1	1	1		10	1	1
												S-CVI/Ave	0.98	
Proportion relevance	1	1	1	1	1	1	0.85	1	1	1		S-CVI/UA		0.85
Average percentage of items deemed pertinent by ten experts											0.98			

Note: S-CVI/Ave: Scale-level Content Validity Index by the averaging method. I-CVI: Item-level Content Validity Index. S-CVI/UA: Scale-level Content Validity Index by the universal agreement method.

**Table 2:** The eigenvalue results of the 16 items (*n* = 365).

Factors	Initial Eigenvalues			Extraction Sums of Squared Loadings			Rotation Sums of Squared Loadings		
	Total	% of Variance	Cumulative %	Total	% of Variance	Cumulative %	Total	% of Variance	Cumulative %
1	8.101	50.633	50.633	7.745	48.408	48.408	3.706	23.161	23.161
2	1.896	11.848	62.481	1.545	9.653	58.061	3.32	20.747	43.908
3	1.337	8.354	70.835	1.003	6.269	64.33	3.268	20.422	64.33
4	0.787	4.92	75.755						
5	0.515	3.22	78.975						
6	0.452	2.823	81.798						
7	0.411	2.571	84.37						
8	0.384	2.401	86.771						
9	0.366	2.289	89.06						
10	0.326	2.04	91.1						
11	0.309	1.93	93.03						
12	0.262	1.638	94.668						
13	0.243	1.517	96.185						
14	0.225	1.408	97.593						
15	0.2	1.253	98.846						
16	0.185	1.154	100						

Extraction method: principal axis factoring.

**Table 3:** Results of factor rotation using Varimax (*n* = 365).

Items	Factor		
	1	2	3
1	0.138	0.204	0.778
2	0.154	0.265	0.717
3	0.194	0.175	0.807
4	0.301	0.175	0.692
5	0.542	0.135	0.489
6	0.605	0.126	0.482
7	0.758	0.146	0.391
8	0.675	0.252	0.329
9	0.671	0.405	0.118
10	0.628	0.4	0.156
11	0.642	0.472	0.066
12	0.548	0.539	0.106
13	0.351	0.679	0.234
14	0.293	0.703	0.282
15	0.232	0.791	0.207
16	0.157	0.782	0.237

Extraction method: principal axis factoring. Rotation method: Varimax with Kaiser normalization. Rotation converged in eight iterations.

**Table 4:** Intraclass correlation coefficient values for the Clinical Reasoning Scale—Arabic (*n* = 30).

Measures	ICC	Confidence Interval 95%		F Test for ICC			*p*-Value
		Lower Bound	Upper Bound	Value	df1	df2	
Single measures	0.949 a	0.896	0. 985	38.50	29	29	<0.001
Average measures	0.974 c	0.945	0.987	38.40	29	29	<0.001

ICC: Intraclass Correlation Coefficient; *p*-value: <0.05 is significant. Single measures ICC refers to the reliability of a single administration of the instrument. Average measures ICC refers to the reliability of the mean score across repeated administrations.; F = F-test statistic; a = Single Measures ICC; c = Average Measures ICC.

**Table 5:** Descriptive statistics of the Clinical Reasoning Scale—Arabic version (*n* = 365).

Items	Mean	SD
Awareness of clinical cues		
I can notice the patient’s needs when I get in contact with the patient.	4.10	0.69
I can notice the patient’s potential health concerns based on the clinical cues I have observed.	4.04	0.69
I can use various data collection methods (such as medical history, physical assessment) to collect clues pertinent to the problem	4.09	0.69
My clinical practical experience can help me to detect a patient’s health concerns.	4.06	0.66
Confirmation of clinical problems		
I can collect all the data on an abnormality before I confirm a patient’s health problems.	3.81	0.75
I can explain the connection between observed cues and a patient’s health problems.	3.86	0.73
I can identify a patient’s health problems by synthesizing the cues collected.	3.87	0.71
I can use theories and nursing knowledge to interpret clinical cues to determine a patient’s health problems.	3.91	0.75
Determination and implementation of actions		
I can think through problem-solving steps before resolving patient issues.	4.0	0.73
I can set a goal for problem-solving based on a patient’s condition.	3.98	0.72
I can find the most appropriate solution based on a patient’s condition.	3.99	0.70
I can provide theory- and evidence-based nursing interventions	4.0	0.71
Evaluation and reflection		
I can evaluate whether a patient’s problems are resolved.	4.13	0.71
I can evaluate effectiveness of problem solving from a variety of aspects.	4.09	0.69
I can re-evaluate a patient’s needs if the problem is not resolved.	4.18	0.70
I can reflect on the steps of problem solving for improvement whether the problem is resolved or not.	4.18	0.69

**Table 6:** Domain-wise scores of the Arabic Clinical Reasoning Scale (CRS-A) (*n* = 365).

Domain-Wise Scores of Clinical Reasoning Scale—Arabic Version				
	Minimum	Maximum	Mean	Std. Deviation
Awareness of clinical cues	2.00	5.00	4.08	0.59
Confirmation of clinical problems	1.75	5.00	3.86	0.64
Determination and implementation of actions	1.75	5.00	3.99	0.62
Evaluation and reflection	1.75	5.00	4.15	0.61

**Table 7:** Comparison of clinical reasoning scores by gender (*n* = 365).

	Gender			CR Score		*t*-Test	*p*-Value
		Frequency	%	Mean	SD		
CRS Scores	Male	68	19	65.86	10.11	1.21	0.23
	Female	297	81	64.12	7.80		

Significance level: *p*-value < 0.05.

**Table 8:** Comparison of clinical reasoning scores by age group (*n* = 365).

	Age Group	Frequency	%	CR Score		F-Test	*p*-Value
				Mean	SD		
CRS Scores	18–23 Years	179	49.04	65.26	8.28	2.08	0.102
	24–29 Years	96	26.30	62.76	8.47		
	30–35 Years	62	16.99	64.03	6.97		
	>35 years	28	7.67	63.86	6.80		

Significance level: *p*-value < 0.05.

**Table 9:** Comparison of clinical reasoning scores by academic performance (*n* = 365).

	CGPA	Frequency	%	CR Score		F-Test	*p*-Value
				Mean	SD		
CRS Scores	Excellent (4.75–5)	42	11.5	65.54	7.37	6.19	<0.001
	Very good (3.75–4.74)	219	60.0	65.31	7.73		
	Good (2.75–3.74)	97	26.6	61.34	8.33		
	Satisfactory (2–2.74)	7	1.9	65.42	9.55		

η^2^ (eta-squared) = 0.049, 95% CI (0.011, 0.092); significance level: *p*-value < 0.05.

**Table 10:** Names of the factors and the items of the Arabic Clinical Reasoning Scale.

Factor/Name	Items
1. Confirmation of clinical problems and determination and implementation of actions	5. I can collect all the data on an abnormality before I confirm a patient’s health problems. يمكنني جمع كافة البيانات عن أي حالة غير طبيعية قبل التأكد من المشاكل الصحية للمريض.
	6. I can explain the connection between observed clues and a patient’s health problems. أستطيع أن أشرح العلاقة بين الاعراض والعلامات الملاحظة والمشاكل الصحية للمريض.
	7. I can identify a patient’s health problems by synthesizing the clues collected. يمكنني التعرف على المشاكل الصحية للمريض من خلال تحليل القرائن التي جمعتها.
	8. I can use theories and nursing knowledge to interpret clinical clues to determine a patient’s health problems. يمكنني استخدام النظريات ومعلومات التمريض لتفسير القرائن السريرية لتحديد المشكلات الصحية للمريض.
	9. I can think through the problem-solving steps before resolving patient issues. يمكنني التفكير من خلال استخدام خطوات حل المشكلات قبل حل مشكلات المرضى.
	10. I can set a goal for problem solving based on a patient’s condition. يمكنني تحديد هدف لحل المشكلات بناءً على حالة المريض
	11. I can find the most appropriate solution based on a patient’s condition. يمكنني العثور على الحل الأنسب بناءً على حالة المريض.
	12. I can provide theory- and evidence-based nursing interventions. يمكنني تقديم تدخلات تمريضية قائمة على النظرية والأدلة.
2. Evaluation and reflection	13. I can evaluate whether a patient’s problems are resolved. يمكنني تقييم ما إذا كانت مشاكل المريض قد تم حلها أم لا.
	14. I can evaluate effectiveness of problem solving from a variety of aspects. يمكنني تقييم فعالية حل المشكلة من جوانب مختلفة
	15. I can re-evaluate a patient’s needs if the problem is not resolved. يمكنني إعادة تقييم احتياجات المريض إذا لم يتم حل المشكلة.
	16. I can reflect on the steps of problem solving for improvement whether the problem is resolved or not. يمكنني التفكير في خطوات حل المشكلات للتحسين سواء تم حل المشكلة أم لا
3. Awareness of clinical cues	1. I can notice patient’s needs when I get in contact with the patient. أستطيع أن ألاحظ احتياجات المريض عندما أتواصل معه.
	2. I can notice patient’s potential health concerns based on the clinical clues I have observed. أستطيع أن ألاحظ المخاوف الصحية المحتملة للمريض بناءً على القرائن السريرية التي لاحظتها
	3. I can use various data collection methods (such as medical history, physical assessment) to collect clues pertinent to the problem. لجمع الأدلة ذات الصلة بالمشكلة الصحية (مثل التاريخ الطبي والتقييم البدن) يمكنني استخدام طرق مختلفة لجمع البيانات
	4. My clinical practical experiences can help me to detect a patient’s health concerns. يمكن أن تساعدني تجاربي العملية السريرية على اكتشاف مخاوف المريض الصحية.

## Data Availability

The data presented in the study are available upon reasonable request from the corresponding author.

## Supplementary Materials

The following supporting information can be downloaded at: https://www.mdpi.com/article/10.3390/nursrep16070214/s1, Supplementary File S1: English version of the Clinical Reasoning Scale; Supplementary File S2: Arabic version of the Clinical Reasoning Scale.

## Public Involvement Statement

No public involvement in any aspects of this research.

## Guidelines and Standards Statement

This manuscript was drafted using the “Strengthening the Reporting of Observational Studies in Epidemiology (STROBE)” for reporting observational studies.

## Abbreviations

The following abbreviations are used in the manuscript:

CRS	Clinical Reasoning Scale
CRS-A	Arabic Clinical Reasoning Scale
CGPA	Cumulative Grade Point Average
CV	Content Validity
I-CVI	Item-wise Content Validity Index
ICC	Intraclass Correlation Coefficient
S-CVI/Ave	Scale-wise Content Validity Index by average method
S-CVI/UA	Scale-wise Content Validity Index by universal agreement method
