# Supplementary material for: Cross-Cultural Adaptation and Psychometric Evaluation of the Arabic Clinical Reasoning Scale Among Nursing Students
Source: Nurs Rep. 2026 Jun 25;16(7):214. doi: 10.3390/nursrep16070214 (PMC13415129; doi:10.3390/nursrep16070214)
Supplement: Supplementary file 1 [file nursrep-16-00214-s001.zip › nursrep-4352256-supplementary S1.pdf]

CLINICAL REASONING SCALE (CRS)

Instructions: This scale is a series of statements to analyze your clinical reasoning ability for nursing process application. There are no right or wrong answers. You will probably agree with some of the statements and disagree with others. Please indicate your own personal feelings about each statement below by marking the numbers that best describe your attitude or beliefs. Please be truthful and describe your attitude, as it really is, not what you would like it to be.

Note. Score: 1 = strongly disagree, 2= disagree, 3= neutral, 4= agree, and 5 = strongly agree.

No.	**Item**	1	2	3	4	5
**Awareness of clinical cues**						
1	I can notice patient's needs when I get in contact with the patient.					
2	I can notice patient's potential health concerns based on the clinical clues I have observed.					
3	I can use various data collection methods (such as medical history, physical assessment) to collect clues pertinent to the problem.					
4	My clinical practical experiences can help me to detect a patient's health concerns.					
**Confirmation of clinical problems**						
5	I can collect all the data on an abnormality before I confirm a patient's health problems.					
6	I can explain the connection between observed clues and a patient's health problems.					
7	I can identify a patient's health problems by synthesizing the clues collected.					
8	I can use theories and nursing knowledge to interpret clinical clues to determine a patient's health problems.					
**Determination and implementation of actions**						
9	I can think through the problem-solving steps before resolving patient issues.					
10	I can set a goal for problem solving based on a patient's condition.					
11	I can find the most appropriate solution based on a patient's condition.					
12	I can provide theory- and evidence-based nursing interventions.					
**Evaluation and reflection**						
13	I can evaluate whether a patient's problems are resolved.					
14	I can evaluate effectiveness of problem solving from a variety of aspects.					
15	I can re-evaluate a patient's needs if the problem is not resolved.					
16	I can reflect on the steps of problem solving for improvement whether the problem is resolved or not.					
