# Supplementary material for: Cross-Cultural Adaptation and Psychometric Evaluation of the Arabic Clinical Reasoning Scale Among Nursing Students
Source: Nurs Rep. 2026 Jun 25;16(7):214. doi: 10.3390/nursrep16070214 (PMC13415129; doi:10.3390/nursrep16070214)
Supplement: Supplementary file 1 [file nursrep-16-00214-s001.zip › nursrep-4352256-supplementary S2.pdf]

(CRS) مقياس الاستدلال السريري لطلاب التمريض

التعليمات: هذا المقياس هو سلسلة من العبارات لتحليل قدرتك على التفكير السريري لتطبيق عملية التمريض. لا توجد اجابات صحيحة أو خاطئة. من المحتمل أن تتفق مع بعض العبارات ولا تتفق مع بعضها. يرجى الإشارة إلى مشاعرك الشخصية حول كل بيان أدناه من خلال تحديد الأرقام التي تصف موقفك أو معتقداتك بشكل أفضل. يرجى أن تكون موضوعيا وصف موقفك كما هو حقا، وليس ما تريده أن يكون.

ملاحظه. النتيجة: 1 = لا أوافق بشدة، 2 = لا أوافق، 3 = محايد، 4 = أوافق، و5 = أوافق بشدة

No.	Item	1	2	3	4	5
	**الوعي بالإشارات السريرية**					
1	.أستطيع أن ألاحظ احتياجات المريض عندما أتواصل معه					
2	**أستطيع أن ألاحظ المخاوف الصحية المحتملة للمريض بناءً على القرائن السريرية التي لاحظتها**					
3	**يمكنني استخدام طرق مختلفة لجمع البيانات (مثل التاريخ الطبي والتقييم البدن) لجمع الأدلة ذات الصلة بالمشكلة الصحية**					
4	**يمكن أن تساعدني تجاربي العملية السريرية على اكتشاف مخاوف المريض الصحية.**					
	**تأكيد المشاكل السريرية**					
5	**يمكنني جمع كافة البيانات عن أي حالة غير طبيعية قبل التأكد من المشاكل الصحية للمريض.**					
6	**أستطيع أن أشرح العلاقة بين الاعراض والعلامات الملاحظة والمشاكل الصحية للمريض.**					
7	**يمكنني التعرف على المشاكل الصحية للمريض من خلال تحليل القرائن التي جمعتها.**					
8	**يمكنني استخدام النظريات ومعلومات التمريض لتفسير القرائن السريرية لتحديد المشكلات الصحية للمريض.**					
	**تحديد وتنفيذ الإجراءات**					
9	**يمكنني التفكير من خلال استخدام خطوات حل المشكلات قبل حل مشكلات المرضى.**					
10	**يمكنني تحديد هدف لحل المشكلات بناءً على حالة المريض**					
11	**.يمكنني العثور على الحل الأنسب بناءً على حالة المريض**					
12	**.يمكنني تقديم تدخلات تمريضية قائمة على النظرية والأدلة**					
	**التقييم والتفكير**					
13	**.يمكنني تقييم ما إذا كانت مشاكل المريض قد تم حلها أم لا**					
14	**يمكنني تقييم فعالية حل المشكلة من جوانب مختلفة**					
15	**.يمكنني إعادة تقييم احتياجات المريض إذا لم يتم حل المشكلة**					
16	**يمكنني التفكير في خطوات حل المشكلات للتحسين سواء تم حل المشكلة أم لا**					
